# Spatial regulation of bone morphogenetic proteins (BMPs) in postnatal articular and growth plate cartilage

**DOI:** 10.1371/journal.pone.0176752

**Published:** 2017-05-03

**Authors:** Presley Garrison, Shanna Yue, Jeffrey Hanson, Jeffrey Baron, Julian C. Lui

**Affiliations:** 1 Section on Growth and Development, Eunice Kennedy Shriver National Institute of Child Health and Human Development, National Institutes of Health, Bethesda, Maryland, United States of America; 2 Laser Capture Microdissection Core Facility, Laboratory of Pathology, National Cancer Institute (NCI), NIH, Bethesda, Maryland, United States of America; University of Colorado Boulder, UNITED STATES

## Abstract

Articular and growth plate cartilage both arise from condensations of mesenchymal cells, but ultimately develop important histological and functional differences. Each is composed of three layers—the superficial, mid and deep zones of articular cartilage and the resting, proliferative and hypertrophic zones of growth plate cartilage. The bone morphogenetic protein (BMP) system plays an important role in cartilage development. A gradient in expression of BMP-related genes has been observed across growth plate cartilage, likely playing a role in zonal differentiation. To investigate the presence of a similar expression gradient in articular cartilage, we used laser capture microdissection (LCM) to separate murine growth plate and articular cartilage from the proximal tibia into their six constituent zones, and used a solution hybridization assay with color-coded probes (nCounter) to quantify mRNAs for 30 different BMP-related genes in each zone. In situ hybridization and immunohistochemistry were then used to confirm spatial expression patterns. Expression gradients for Bmp2 and 6 were observed across growth plate cartilage with highest expression in hypertrophic zone. However, intracellular BMP signaling, assessed by phospho-Smad1/5/8 immunohistochemical staining, appeared to be higher in the proliferative zone and prehypertrophic area than in hypertrophic zone, possibly due to high expression of Smad7, an inhibitory Smad, in the hypertrophic zone. We also found BMP expression gradients across the articular cartilage with BMP agonists primarily expressed in the superficial zone and BMP functional antagonists primarily expressed in the deep zone. Phospho-Smad1/5/8 immunohistochemical staining showed a similar gradient. In combination with previous evidence that BMPs regulate chondrocyte proliferation and differentiation, the current findings suggest that BMP signaling gradients exist across both growth plate and articular cartilage and that these gradients may contribute to the spatial differentiation of chondrocytes in the postnatal endochondral skeleton.

## Introduction

Long bones form initially from mesenchymal condensates which differentiate into cartilage. Later in development, primary and secondary ossification centers form and expand, converting most of the structure into bone but leaving two types of cartilage—growth plate and articular cartilage [1–3]. Thus the postnatal growth plate cartilage and articular cartilage share a common origin but subsequently follow distinct developmental paths, which lead to important differences in structure and function. The molecular mechanisms that underlie the similarities and differences between growth plate and articular cartilage are poorly understood.

The postnatal growth plate cartilage is located near the ends of long bones, peripheral to the primary ossification center but central to the secondary ossification center. The primary function of the growth plate is to cause bone elongation by a two-step process that involves chondrogenesis followed by endochondral ossification. The growth plate is composed of three histologically and functionally distinct zones of chondrocytes, the resting zone (RZ), proliferative zone (PZ) and hypertrophic zone (HZ). The RZ lies closest to the secondary ossification center in the epiphysis. The RZ chondrocytes serve as progenitor cells which give rise to the cells of the PZ and HZ and also direct the cellular orientation of the growth plate [4]. In the PZ, clones of chondrocytes are arrayed in columns parallel to the long axis of the bone. In this zone, the chondrocytes proliferate rapidly. Nearing the metaphysis, the cells stop dividing and enlarge, forming the HZ. At the bottom of the hypertrophic zone, the terminally hypertrophic chondrocytes may either directly transdifferentiate into osteoblast [5], or undergo apoptosis. The area is then invaded by blood vessels, osteoblasts and osteoclasts from the primary ossification center, which remodel the hypertrophic cartilage into bone [6].

The articular cartilage is located peripheral to the secondary ossification center, lining the joint surface. The articular cartilage facilitates load transmission while minimizing friction by providing a smooth, lubricated surface for articulation. Like growth plate, articular cartilage is composed of three zones of chondrocytes, the superficial zone (SZ), mid zone (MZ), and deep zone (DZ). The cells of the SZ are flat, tightly spaced, and oriented parallel to the surface of the bone. The SZ chondrocytes are exposed to the synovial cavity. In the MZ and DZ, the cells are increasingly large and separated by extracellular matrix. In addition to its mechanical role at the joint surface, articular cartilage is also responsible for radial growth of the epiphysis during juvenile life. Fate-mapping studies suggest that the slowly cycling cells of the SZ serve as a progenitor population for the deeper layers of the articular cartilage [7], analogous to the role of the RZ of the growth plate.

Bone morphogenetic proteins (BMPs) are important regulators of bone and cartilage cell proliferation and differentiation [8]. BMPs belong to the TGF-β superfamily of paracrine factors and act by binding to specific cell surface serine-threonine kinase receptors, leading to phosphorylation of SMADs, which in turn act as transcription factors. Prior studies suggest the presence of a BMP signaling gradient across the growth plate cartilage, with BMP antagonists expressed primarily in the RZ and BMP ligands expressed primarily in the HZ [8, 9]. There is evidence suggesting this gradient helps direct the differentiation of RZ chondrocytes to PZ chondrocytes and then to HZ chondrocytes [10, 11].

Because chondrocytes in the articular cartilage, like those in the growth plate cartilage, undergo progressive differentiation from progenitor cells to form distinct spatial zones, we hypothesized that a BMP gradient similar to that found in the growth plate may also be present across the zones of the articular cartilage. To test this hypothesis and directly compare BMP signaling present in the growth plate and articular cartilage, we used laser capture microdissection (LCM) to separate murine growth plate and articular cartilage from the proximal tibia into their six constituent zones. We then used a solution hybridization assay with color-coded probes (nCounter Analysis, Nanostring Technologies, Seattle, WA) to quantify mRNAs for 30 different BMP-related genes in each zone and to characterize the changes in the BMP pathway as the chondrocytes undergo spatial differentiation in both types of cartilage. *In situ* hybridization and immunohistochemistry were then used as confirmatory approaches.

## Materials and methods

### Animal procedures

All animal use described in this study was approved by the National Institute of Child Health and Human Development Animal Care and Use Committee. One-week-old C57BL/6 male mice (Charles River Laboratory, Wilmington, MA) were euthanized by decapitation and proximal tibias were excised. For the solution hybridization assay, excised cartilage was immediately embedded in optimum cutting temperature compound (OCT, Electron Microscopy Sciences, Hatfield, PA), frozen on dry ice, and stored at -80°C until subsequently used for LCM. For *in situ* hybridization and immunohistochemistry, cartilage was fixed overnight in 2% (v/v) paraformaldehyde (Electron Microscopy Sciences) and 0.2% (v/v) glutaraldehyde (Electron Microscopy Sciences) at 4°C, then decalcified in 10% (v/v) EDTA and 0.5% (v/v) paraformaldehyde, pH 7.4, and subsequently embedded in paraffin for sectioning.

### Laser capture microdissection of articular and growth plate cartilage

Frozen longitudinal cartilage sections (10 μm) were made using a cryostat (Leica Biosystems, St. Louis, MO) and placed on PEN membrane frame slides (Applied Biosystems, Foster City, CA) precoated with 0.01% (w/v) poly-D-lysine (Sigma-Aldrich, St. Louis, MO). Slides were kept at -80°C for short-term storage. Slides were thawed and stained as previously described [12] with the following modifications: 70% ethanol, 30 s; deionized water, 30 s; Mayer’s hematoxylin (Sigma-Aldrich), 30 s; deionized water, 15 s; 70% ethanol, 15 s; eosin Y (Sigma-Aldrich), 1 s; 95% ethanol, 15 s; 100% ethanol, 15 s; xylene (Sigma-Aldrich), 30 s. Staining was optimized to shorten staining time and minimize tissue detachment. Articular cartilage zones (SZ, MZ, and DZ) and growth plate zones (HZ, PZ, and RZ) were defined as follows: SZ, a two-cell layer, consist of only flat chondrocytes on the articular surface; MZ, small, round chondrocytes below the superficial layer; DZ, chondrocytes below the MZ, beginning to hypertrophy but above the future secondary ossification center, which consist of hypertrophic chondrocytes; RZ, small, round chondrocytes below the future secondary ossification center, but above the proliferative columns; PZ, proliferative columns between RZ and prehypertrophic zone, which contains chondrocytes beginning to hypertrophy; HZ, hypertrophic chondrocytes below the pre hypertrophic zone and above the trabecular bone. Zones were microdissected using the cut and capture method [13] in the Veritas LCM system (Applied Biosystems) using CapSure HS Caps (Applied Biosystems) and dissolved in tissue extraction buffer (Applied Biosystems, PicoPure RNA Isolation Kit) for 30 minutes at 42°C in a hybridization oven (Thermo Scientific, Waltham, MA). For each zone, tissue dissected from 40 sections from a single animal was pooled, and RNA isolation was performed using the PicoPure RNA Isolation Kit (Thermo Scientific). RNA concentrations were assessed by spectrophotometry (NanoDrop, Wilmington, DE). 3 to 8 ng of total RNA was obtained from each zone.

### Quantitation of mRNAs by solution hybridization

mRNAs of interest were quantified by multiplex solution hybridization assay. Our mRNA samples were obtained from proximal tibias of 1-wk-old male mice (n = 6). From individual animals, 6 different cartilage zones (SZ, MZ, DZ, RZ, PZ, HZ) were dissected, resulting in a total of 36 samples. Each mRNA sample was hybridized to a mixture of 54 different probes, each of which was uniquely identified with a molecular barcode (a string of fluorescent dyes), and then the hybridized probes were quantified by microscopic imaging. The technique does not require reverse transcription or DNA amplification and provides high reproducibility and sensitivity for the detection of multiple transcripts [14–16]. A custom probe set for 30 BMP-related genes (S1 Table), one cartilage marker (Col2a1), 5 cartilage zonal markers, and four housekeeping genes was designed by NanoString Technologies, based on the mouse (*Mus musculus*) sequences in the NCBI RefSeq database. In addition to the target-specific probe set, the kit includes six positive probes for quality control and eight negative controls whose sequences were obtained from the External RNA Controls Consortium and are confirmed not to hybridize with mammalian RNA. 50 ng of RNA from each sample underwent hybridization and quantitation at the Center for Cancer Research (CCR) Genomics Core. RNA counts were normalized to the geometric mean of four housekeeping genes: *Actb*, *Hmgn1*, *Rpl13a*, and *Rpl35*. Background correction was performed as follows: the mean of the negative controls plus two standard deviations was used as a threshold value for transcript detection; any value below this threshold value of 25 was set to 25.

### *In situ* hybridization

cDNA from 1-wk-old mouse growth plate was amplified using Phusion DNA polymerase (Thermo Scientific) with primers that contained either a T7 promoter (for sense probes) or an SP6 promoter (for antisense probes) (S2 Table). Single-stranded digoxigenin-labeled riboprobes for *in situ* hybridization were transcribed using DIG RNA Labeling Kit (Roche Diagnostics, Indianapolis, IN) following the manufacturer’s protocol. Riboprobes were purified by Micro Bio-Spin Columns P-30 Tris RNase free (Bio-Rad, Hercules, CA), followed by alkaline hydrolysis for 30 min as previously described [17]. Paraffin-embedded sections of 1-wk-old mouse knee joint epiphyseal cartilage were hybridized to digoxigenin-labeled riboprobes, as described by Bandyopadhyay *et al*. and Murtaugh *et al*. [18, 19] with some modifications. Notably, tissues were permeabilized with 300 mg/ml of proteinase K (Thermo Scientific) at 37°C for 30 min prior to acetylation and prehybridization. For detection, tissue sections were incubated with anti-digoxigenin alkaline phosphatase Fab fragments (Roche Diagnostics) for 2 h at room temperature and then treated with nitro-blue tetrazolium chloride/5-bromo-4-chloro-3’-indolyphosphate p-toluidine salt (NBT/BCIP) (Sigma-Aldrich) in the dark until a colorimetric change was detected. The sections were visualized using a ScanScope CS digital scanner (Aperio Technologies, Vista, CA) under bright field microscopy.

### Immunohistochemistry

Growth plate sections from 1-wk-old male mice knee joints were baked at 65°C for 1 hour, deparaffinized in xylene, rehydrated through ethanol series (100%, 100%, 95%, 70%), and rinsed with PBS. For Bmp2, Bmp6, Grem1, and pSmad1/5/8, antigen retrieval was performed using proteinase K (100μg/ml in PBS, 30 mins). For Smad7, heat-induced antigen retrieval in citric acid buffer (pH6.0) was performed. Endogenous peroxidase activity was blocked by 3% H_2_O_2_. Staining was performed using anti-Bmp2 (abcam, ab14933, 1:250), anti-Bmp6 (abcam, ab15640, 1:100), anti-Grem1 (abcam, ab189267, 1:100), anti-pSmad1/5/8 (Cell Signaling, #13820, 1:200), and anti-Smad7 (ThermoFisher Scientific, PA1-41506, 1:50), with a VECTASTAIN ABC kit (Vector Laboratories, Burlingame, CA) followed by DAB Substrate kit (Vector Laboratories) according to manufacturer’s instructions. For pSmad1/5/8, Tyramide Signaling Amplication (Thermo Scientific) was used instead of VECTASTAIN ABC kit to increase the signal intensity. Sections were mounted without counterstaining or counter stained with methyl green.

### Statistical analysis

Statistical analysis of solution hybridization (Nanostring) data was performed using Partek Genomic Suite 6.6 (Partek Inc., St Louis, MO) after normalization and background correction. One-way ANOVA was used to assess statistical significance for mRNA expression across cartilage zones, followed by pairwise comparisons between zones with correction for multiple comparisons by the Holm-Sidak method.

### Data availability

All supporting data will be made available by the corresponding author upon request.

## Results

### Validation of dissection technique using zonal markers

**Fig 1:**
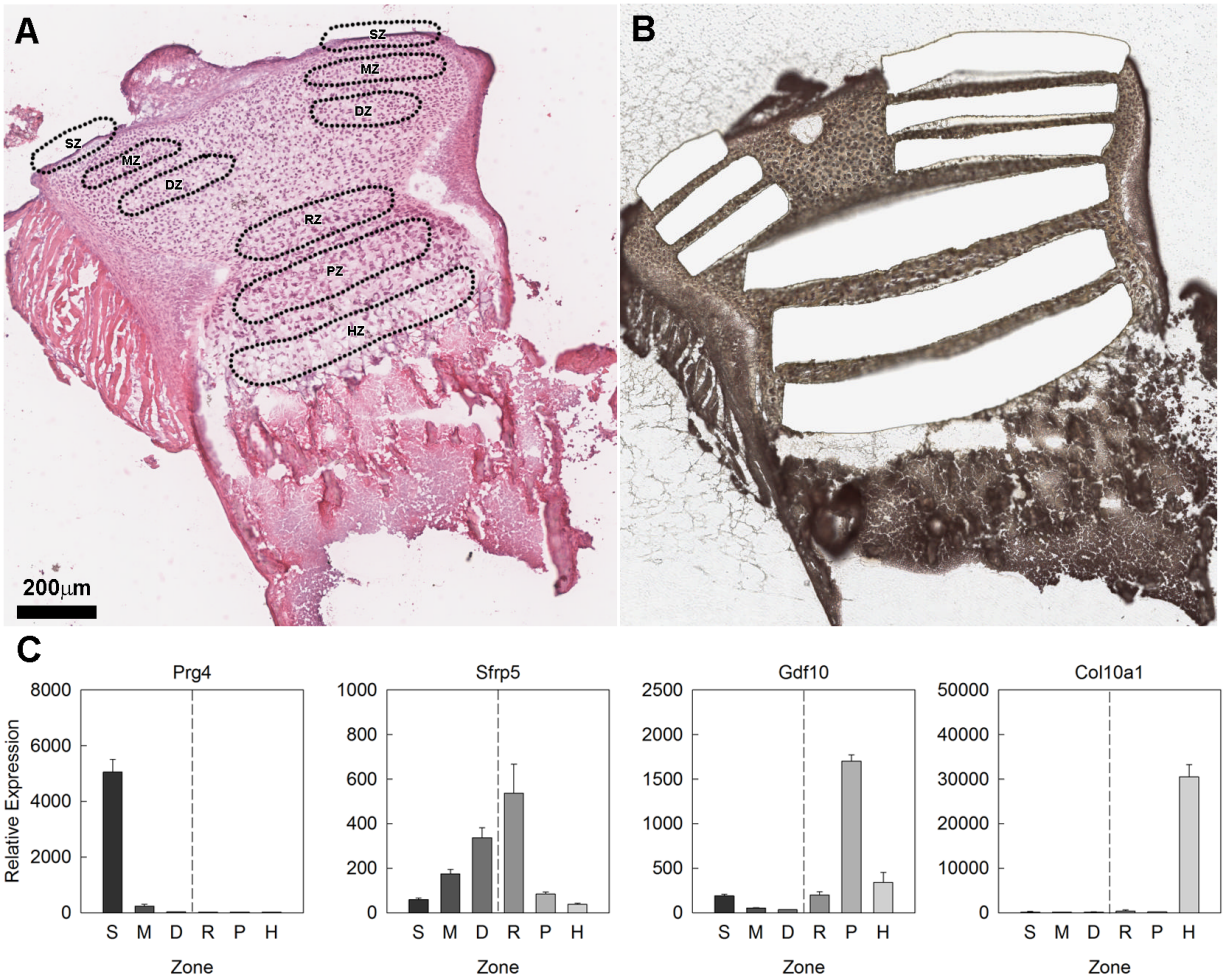
Laser capture microdissection of mouse articular and growth plate cartilage. **(A**) Haemotoxylin & eosin stained section of formalin fixed, decalcified 1-wk old mouse proximal tibial cartilage showing the location of zones in the cartilage that were targeted by laser capture microdissection (LCM). **(B)** Haemotoxylin & eosin stained frozen sections of 1-wk old mouse proximal tibial cartilage after LCM showing regions that were excised for RNA isolation. **(C)** Relative gene expression (mean ± SEM) of zonal markers used for dissection validation. S, superficial zone; M, middle zone; D, deep zone; R, resting zone; P, proliferative zone; H, hypertrophic zone. Scale bar applies to both (A) and (B).

Articular and growth plate cartilage from the proximal tibias of 7-day old mice were dissected using LCM into SZ, MZ, and DZ of the articular cartilage and RZ, PZ, and HZ of the growth plate cartilage (Fig 1a and 1b). mRNAs in each sample were quantified by multiplex solution hybridization assay using probes labeled with fluorescent barcodes (nCounter). This analysis included a broad list of BMP agonists, functional antagonists, receptors, and downstream signaling modulators (SMADs) that are known to be involved in BMP signaling (S1 Table). In addition, we have also included previously identified zonal markers to confirm the accuracy of the dissection (S1 Table). The zonal markers used in this study were Prg4 for SZ, Sfrp5 for DZ and RZ, Gdf10 and Prelp for PZ, and Col10a1 for HZ. Prg4 was expressed at levels 24-fold higher in the SZ than in the adjacent MZ (p<0.001) (Fig 1c). Sfrp5 was expressed at levels 1.9-fold higher in the DZ than in the adjacent MZ (p<0.01) and 5.8-fold higher in the RZ than in the adjacent PZ (p<0.001) (Fig 1c). Gdf10 was expressed at levels 6.5- and 9.4-fold higher in the PZ than in the adjacent HZ and RZ, respectively (both p<0.001) (Fig 1c) Similarly, Prelp was expressed at levels 2.3-and 8.2-fold higher in the PZ than in RZ and HZ respectively (both p<0.001) (S1 Table). Lastly, Col10a1 was expressed at levels 180-fold higher in the HZ than in the adjacent PZ (p<0.001) (Fig 1c).

### BMP expression gradients in growth plate cartilage

**Fig 2:**
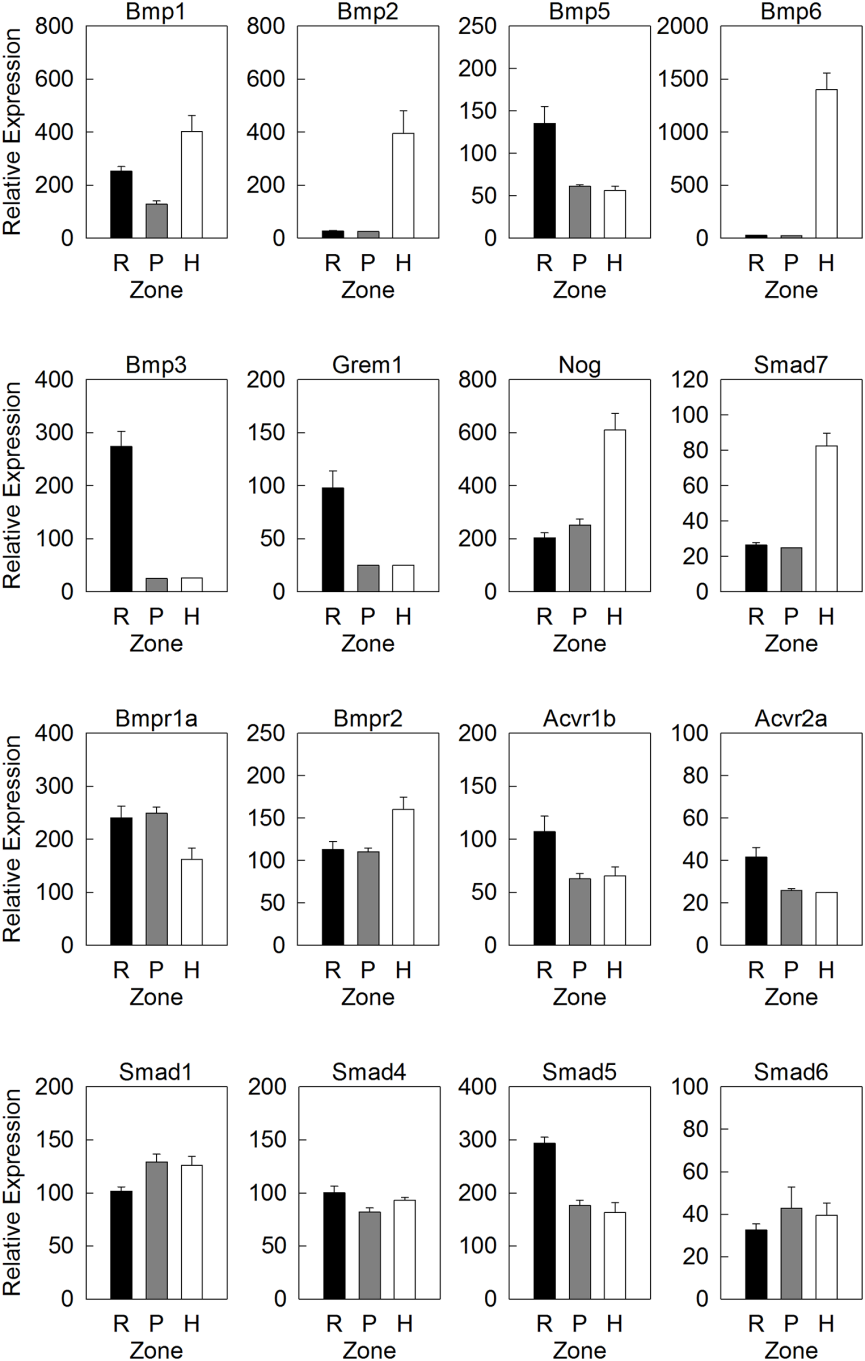
Expression of genes involved in BMP signaling in growth plate cartilage. Relative gene expression (mean ± SEM) of a subset of BMP agonists (top row), BMP functional antagonists (second row), BMP receptors (third row) and downstream signaling modulators (bottom row) in resting zone (R), proliferative zone (P) and hypertrophic zone (H) of 1-wk old mouse tibial growth plate, measured by a multiplex solution hybridization assay (nCounter). A full list of BMP-related gene and expression values were shown in S1 Table.

Solution hybridization data indicated that several BMP agonists were primarily expressed in the HZ: Bmp1 (3.0-fold greater than PZ), Bmp2 (14-fold), and Bmp6 (55-fold) (all p<0.001, Fig 2). Conversely, BMP system functional antagonists, Grem1 and Bmp3, were primarily expressed in the RZ: Grem1 (3.7-fold greater than PZ) and Bmp3 (11-fold) (both p<0.001) (Fig 2). These patterns support previous studies using microarray that a BMP expression gradient exists across the growth plate with greater ligand expression in the HZ and greater antagonist expression in the RZ, although there were also some differential expression patterns that did not fit this general trend. In particular, two BMP antagonists, Nog and Smad7, were more highly expressed in the HZ than in the PZ (Nog, 2.4-fold greater in HZ than PZ; Smad7, 3.3-fold greater in HZ than PZ; both p<0.01) (Fig 2).

Genes encoding BMP receptors (Bmpr1a, Bmpr1b, Acvr1, Acvr1b, Acvrl1, Bmpr2, Acvr2a, Acvr2b) showed only modest differences among zones of the growth plate (Fig 2). Bmpr1b, Acvr1a, and Acvrl1 did not differ significantly among zones. Bmpr1a showed 1.6-fold greater expression in PZ than HZ (p<0.05) (Fig 2). Bmpr2 was expressed at levels 1.5-fold greater in HZ than PZ (p<0.01) (Fig 2). Acvr1b showed greater expression in RZ than PZ (1.7-fold, p<0.05) as did Acvr2a (1.6-fold, p<0.01) (Fig 2). Acvr2b showed the least expression in PZ (1.4-fold greater in RZ than PZ, 1.4-fold greater in HZ than PZ, both p<0.05, S1 Table).

We also studied SMADs, which are important downstream signal transducers and modulators of BMP and TGF-β signaling. We used solution hybridization to assess the expression of Smad1, 5, and 8 (R-SMADs—responsible for mediating BMP signaling) Smad4 (a co-SMAD—dimerizes sith phosphorylated R-SMADs), and Smad6 and 7 (I-SMADs—act as functional antagonists of BMP signaling), Smad7 was the only Smad with a dramatic differential expression across different zones (as described above, 3.3-fold greater in HZ than PZ; p<0.01). The other Smads studied showed only modest differences among zones without a consistent overall pattern. Smad1 was expressed 1.3-fold greater in PZ than RZ (p<0.05), Smad4 was expressed 1.2-fold greater in RZ than PZ (p<0.05), Smad5 was expressed 1.7-fold greater in RZ than PZ (p<0.001) (Fig 2), and Smad6 and Smad8 (S1 Table) showed no significant zonal variation. Smad2 and 3, which are R-SMADs responsible for TGF-β signaling, were not included in our current analysis.

### BMP expression patterns in articular cartilage

**Fig 3:**
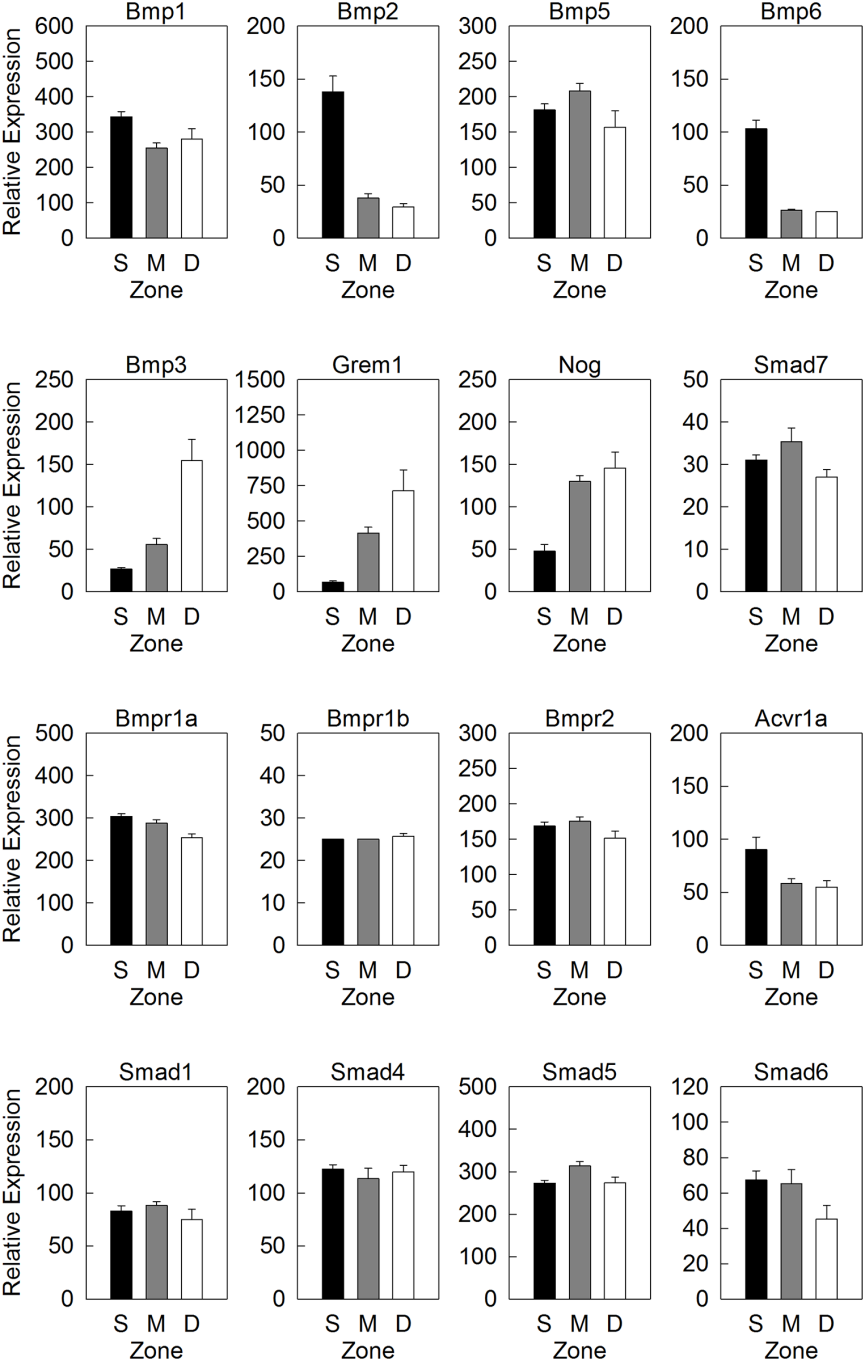
Expression of genes involved in BMP signaling in articular cartilage. Relative gene expression (mean ± SEM) of a subset of BMP agonists (top row), BMP functional antagonists (second row), BMP receptors (third row) and downstream signaling modulators (bottom row) in superficial zone (S), middle zone (M) and deep zone (D) of 1-wk old mouse tibial growth plate, measured by a multiplex solution hybridization assay (nCounter). A full list of BMP-related gene and expression values were shown in S1 Table.

We hypothesized that a BMP expression gradient may also exist across the zones of the articular cartilage. Interestingly, expression of many BMP system agonists was highest in the SZ and MZ: Bmp1 (1.3-fold greater in SZ than MZ, p<0.05), Bmp2 (3.6-fold greater in SZ than MZ, p<0.001), Bmp5 (1.4-fold greater in MZ than DZ, p<0.05) and Bmp6 (3.9-fold greater in SZ than MZ, p<0.001) (Fig 3). In contrast, many BMP system functional antagonists were primarily expressed in the MZ and DZ: Bmp3 (2.7-fold greater in DZ than MZ, 2.0-fold greater in MZ than SZ, both p<0.001), Grem1 (1.6-fold greater in DZ than MZ, 6.4-fold greater in MZ than SZ, both p<0.05), and Nog (2.8-fold greater in MZ than SZ, p<0.001) (Fig 3). Collectively, our data suggests the presence of a BMP gradient across the articular cartilage. However, the direction of the gradient is spatially reversed compared to that of the growth plate, such that BMP agonists were more abundant on the articular surface while antagonists were enriched in the deeper area.

Similar to the growth plate, expression of BMP receptors and SMADs showed only modest differences among zones with no evident overall pattern. Four of eight studied receptors showed no significant zonal variation: Bmpr1a, Bmpr1b, Bmpr2 and Acvr2a (Fig 3 and S1 Table). The remaining receptors did exhibit modest but statistically significant zonal expression variation: Acvrl1 (1.4-fold greater in MZ than DZ, p<0.05), Acvr1a (1.5-fold greater in SZ than MZ, p<0.05), Acvr1b (1.9-fold greater in MZ than SZ, p<0.01), and Acvr2b (1.6-fold greater in MZ than SZ, 1.4-fold greater in MZ than DZ, both p<0.05) (Fig 3 and S1 Table). Smad1, Smad4, Smad5 and Smad6, did not show significant variation in expression among zones (Fig 3). Smad7 showed higher expression in MZ than DZ (1.3-fold, p<0.01) and Smad8 showed highest expression levels in MZ (1.6-fold greater in MZ than SZ, 1.4-fold greater in MZ than DZ, both p<0.05) (Fig 3 and S1 Table).

### Validation of mRNA expression by in situ hybridization

**Fig 4:**
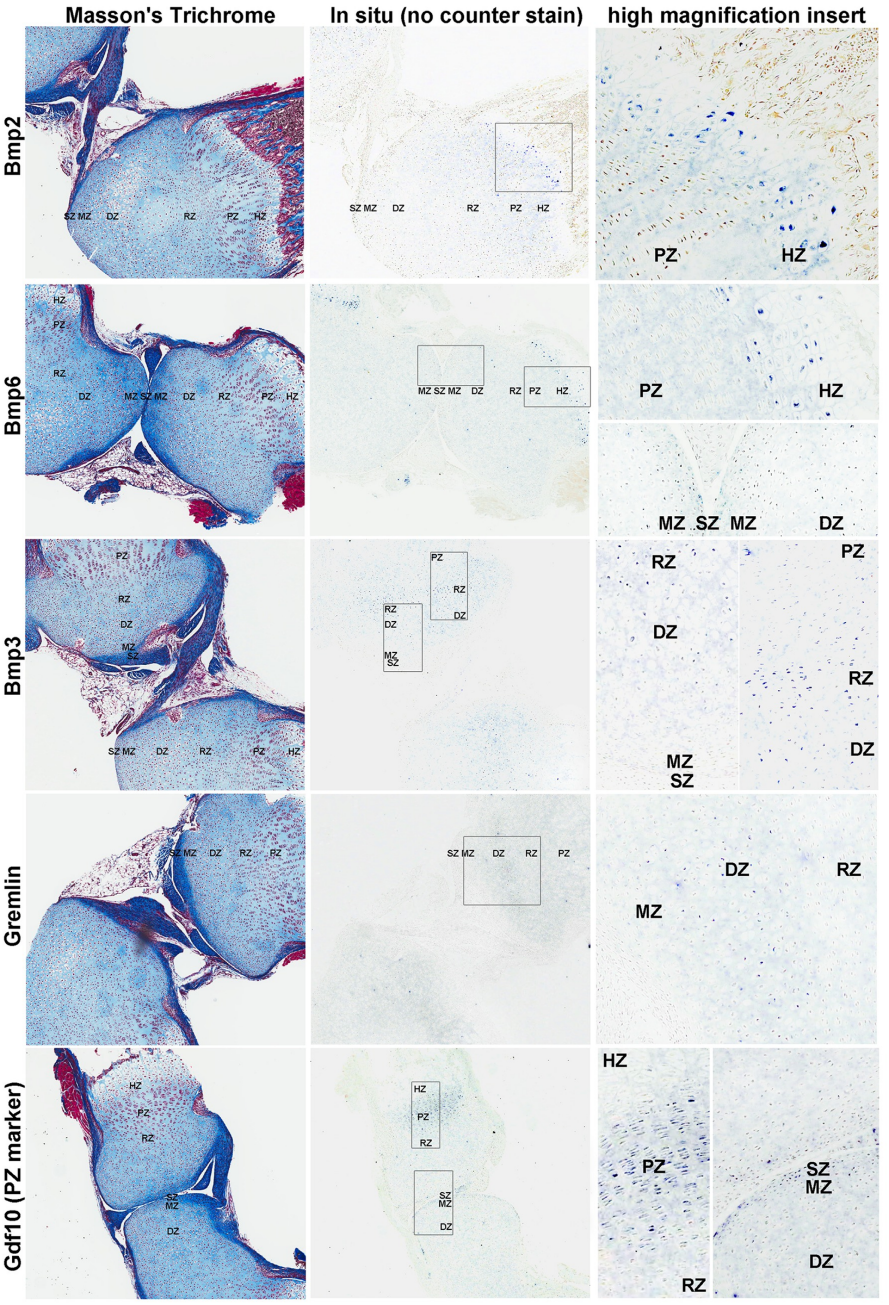
In situ hybridization for BMP-related genes in articular and growth plate cartilage. Formalin fixed, decalcified sections of 1-wk old mouse tibial cartilage were hybridized to DIG-labeled riboprobes, producing a purple color and were visualized by scanning the slides with a ScanScope CS digital scanner using bright field microscopy. Left panel, Mason-trichrome stained sections; middle panel, in situ hybridization without counterstaining; right panel, higher magnification views taken from within the rectangular area indicated in the corresponding middle panel. SZ, superficial zone; MZ, middle zone; DZ, deep zone; RZ, resting zone; PZ, proliferative zone; HZ, hypertrophic zone.

*In situ* hybridization was used to confirm some of the solution hybridization findings. We first performed an *in situ* hybridization for Gdf10, which is a zonal marker previously shown to be expressed in the PZ [20] and found to be very highly expressed in the PZ and slightly elevated in the SZ in our current solution hybridization data (S1 Table). *In situ* hybridization showed that Gdf10 mRNA was strongly expressed in the PZ of the growth plate and also expressed in the SZ of the articular cartilage. This agreement between independent methods helps confirm both the validity of our solution hybridization data and our *in situ* hybridization technique. We next used *in situ* hybridization to study the location of BMP agonists Bmp2 and 6, and BMP antagonists Bmp3 and Gremlin, in the articular and growth plate cartilages. For agonists, Bmp6 was found in both the SZ and HZ (Fig 4), which confirmed our solution hybridization findings, while Bmp2 was detected in the HZ but not the SZ (Fig 4). mRNA for functional antagonists, Bmp3 and Grem1, were detected in the DZ and RZ area, with Bmp3 showing a relatively strong signal in the RZ and Grem1 showing a signal primarily in the DZ (Fig 4), thus validating the pattern found using solution hybridization.

### Assessment of BMP signaling gradients by immunohistochemistry

**Fig 5:**
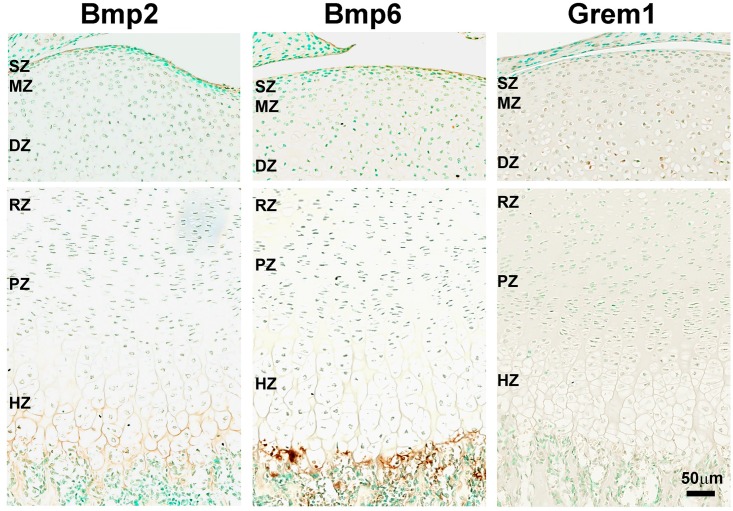
Immunohistochemistry for BMP-related proteins in articular and growth plate cartilage. Formalin fixed, decalcified sections of 1-wk old mouse tibial cartilage were stained with anti-Bmp2, anti-Bmp6, and anti-Grem1 antibody and signals were developed with DAB substrate (brown color). Tissues were counterstained in methyl green and visualized by scanning with a ScanScope CS digital scanner using bright field microscopy. SZ, superficial zone; MZ, middle zone; DZ, deep zone; RZ, resting zone; PZ, proliferative zone; HZ, hypertrophic zone.

We used immunohistochemistry to investigate the spatial expression of BMPs in the articular and growth plate cartilage at the protein level. In agreement with our mRNA findings by solution hybridization and *in situ* hybridization, Bmp2 and 6 proteins were detected in both the HZ and the articular surface (Fig 5). Consistent with the role of these Bmp ligands as autocrine/paracrine growth factors, they were present primarily in the intercellular space. Similarly, we confirmed that Gremlin protein was expression in the DZ of the articular cartilage (Fig 5). Taken together, the spatial distribution of BMP ligands and antagonists that we found by solution hybridization, in situ hybridization, and immunohistochemistry support the concept that BMP signaling gradients exist across both the growth plate cartilage and the articular cartilage, with highest BMP activity in the HZ and SZ.

**Fig 6:**
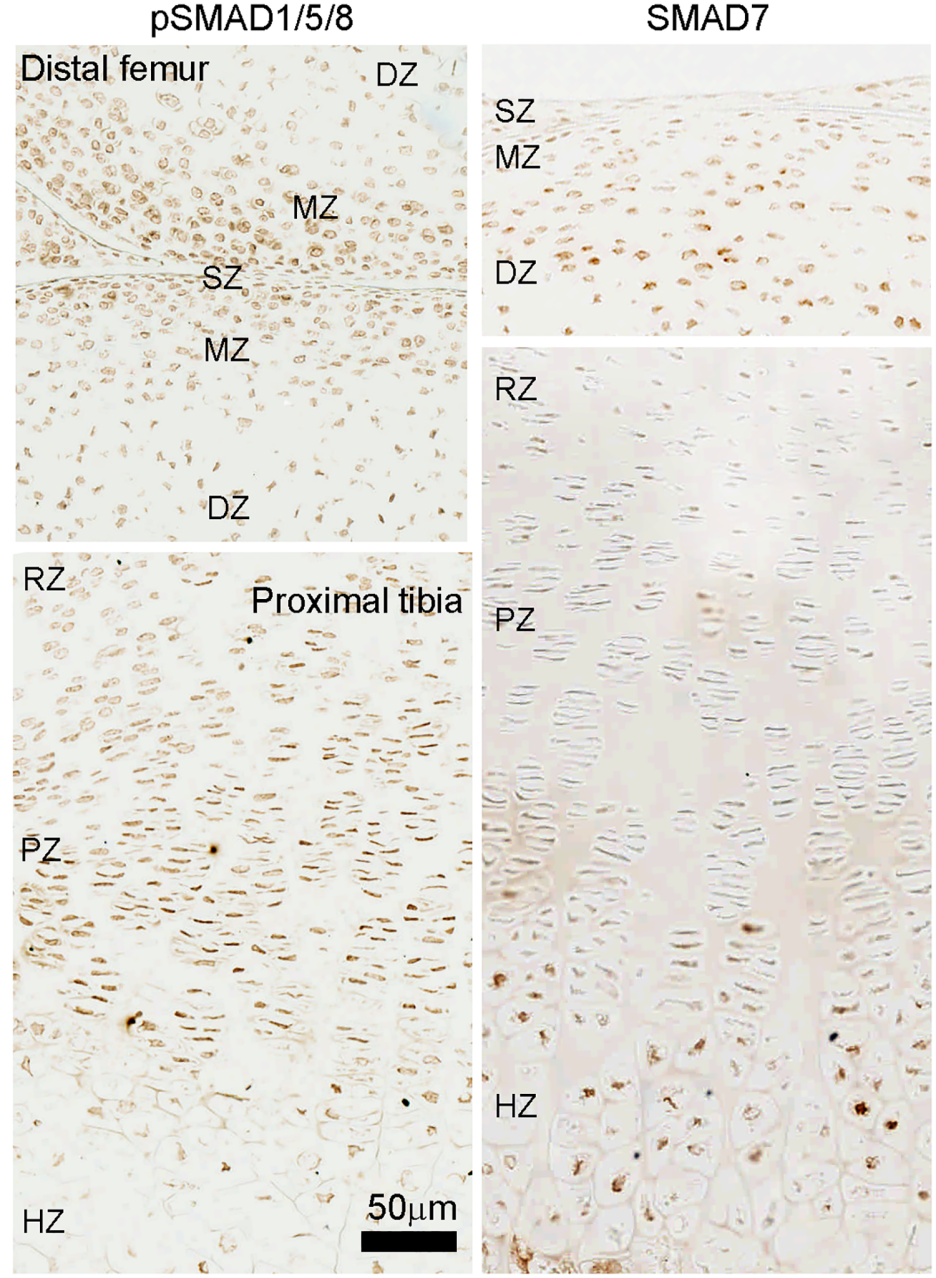
Immunohistochemistry for SMADs in articular and growth plate cartilage. Formalin fixed, decalcified sections of 1-wk old mouse tibial cartilage were stained with anti-pSmad1/5/8 and anti-Smad7 antibody. Signals were developed with DAB substrate (brown color) with no counterstaining. pSmad1/5/8 represents activation of BMP intracellular signaling, while Smad7 is an I-SMAD that functions as a functional antagonist of BMP signaling. SZ, superficial zone; MZ, middle zone; DZ, deep zone; RZ, resting zone; PZ, proliferative zone; HZ, hypertrophic zone.

To further test this hypothesis, we used immunohistochemistry to detect phosphorylation of Smad1/5/8 as a readout of BMP intracellular signaling. Consistent with our hypothesis, the signal for pSmad1/5/8 in the articular cartilage was higher toward the articular surface and decreased in the DZ (Fig 6, left panel). However, surprisingly, we found that in the growth plate, the level of pSmad1/5/8 was highest in the PZ and pre-hypertrophic area, but decreased in the HZ (Fig 6, left panel). These findings suggest that BMP intracellular signaling maybe suppressed in the HZ even though ligands Bmp2 and 6 are most highly expressed in this zone. We next hypothesized that this apparent discrepancy might be explained by the increased expression of the Smad7 (an inhibitory Smad) in the HZ, which we observed by solution hybridization (Fig 2). To confirm this differential expression pattern, we used immunohistochemistry to detect the protein expression of Smad7. Consistent with the solution hybridization mRNA results, we found that Smad7 protein was upregulated in the HZ (Fig 6, right panel). Because Smad7 is an I-SMAD that competes for R-SMAD binding, increased Smad7 expression may help explain the decreased BMP signaling in the HZ. We also found that Smad7 protein was more highly expressed in the DZ (Fig 6, right panel), which is consistent with a higher BMP signaling activity found in the SZ and MZ (Fig 6, left panel).

## Discussion

**Fig 7:**
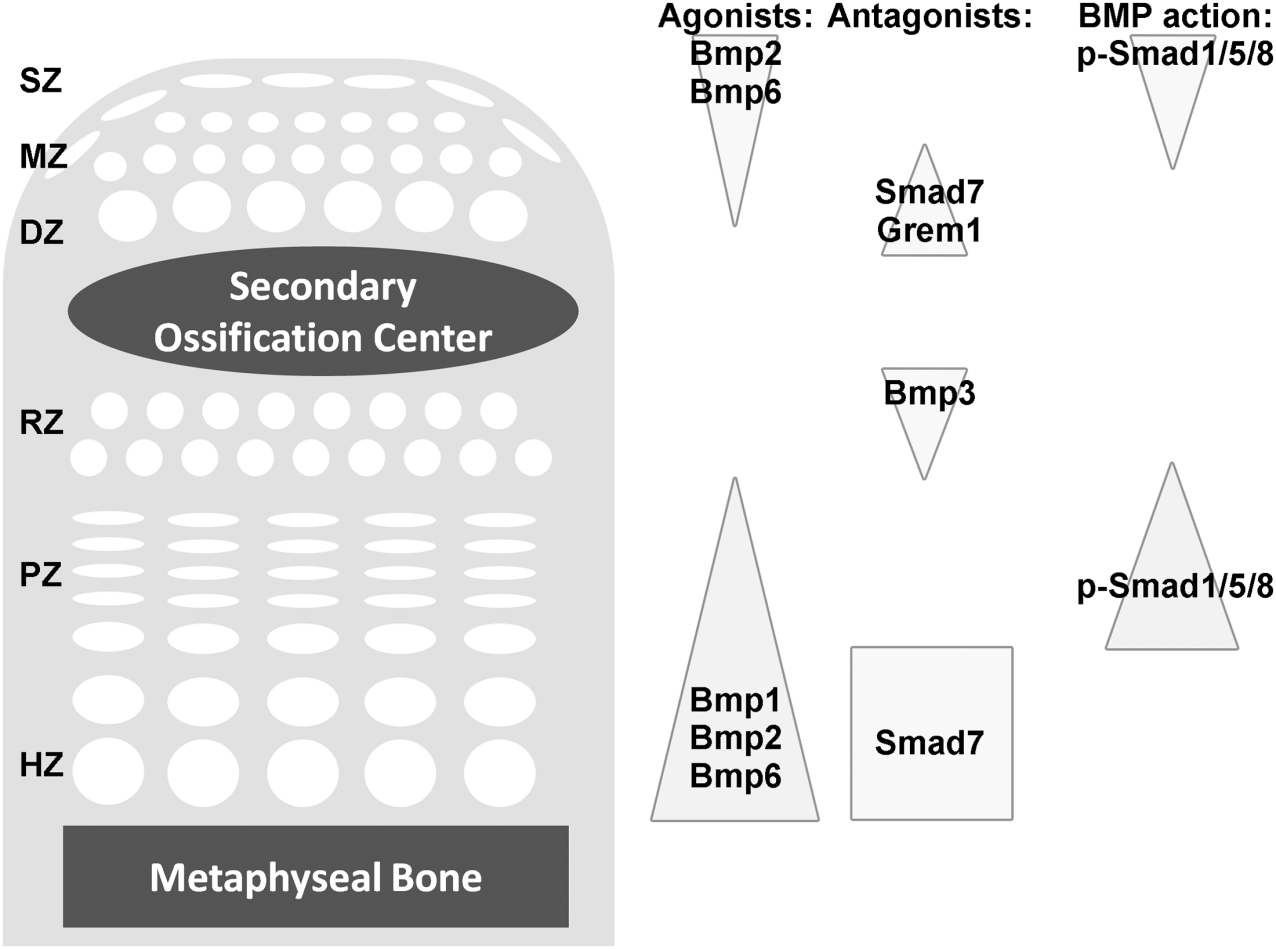
Model of BMP expression gradients in the articular and growth plate cartilage. Our current findings suggest BMP gradients in the articular and growth plate cartilages, with higher BMP signaling on the articular surface and the proliferative zone, and lower BMP signaling in the deep and resting zone. Interestingly, although BMP ligands Bmp2 and 6 were most highly expressed in the HZ, BMP signaling (based on pSmad1/5/8) was higheer in the PZ, perhaps because of high Smad7 expression in HZ. The gene names were placed to indicate regions of high expression.

We used LCM and solution hybridization assay with barcoded probes to assess expression patterns of BMP pathway components in the zones of both growth plate and articular cartilage (Fig 7). Our results confirmed our previous findings in the growth plate cartilage that expression of BMP agonists Bmp2 and Bmp6 were highest in HZ and expression of BMP antagonists Bmp3 and Grem1 were highest in RZ [8]. Additionally, a BMP gradient was identified in articular cartilage with agonists, Bmp2 and Bmp6, expressed primarily in the superficial zone and functional antagonists, Bmp3, Gremlin, and Noggin, expressed primarily in the deep zone. The methodology employed in this study allowed us to make direct comparisons of quantitative levels of expression of specific mRNAs between the zones of articular cartilage and growth plate cartilage. This comparison revealed that, while BMP agonists are expressed in relatively high levels in SZ compared to the remaining articular cartilage, they are expressed in considerably lower levels in the SZ of articular cartilage than the HZ of growth plate.

Isolation of the 7-day-old murine cartilaginous zones was accomplished using LCM. This dissection technique allows for microscopic visualization of cartilage histology and precise tissue selection. However, complete separation of histologic zones is still technically challenging given the size of the tissue sample and the limited layers of cells within each zone. To assess cross contamination between zones, we measured expression of established molecular markers for specific zones and found that these expression levels were consistent with the known expression patterns for these genes. Although some cross contamination between adjacent zones may still occur, such contamination would only decrease the variation in transcript expression between zones, and thus a completely precise isolation of zones would likely demonstrate even stronger differential expression patterns than those observed in our study. Solution hybridization assay using probes with color-coded labels provides an alternative platform to expression microarray and RNA-seq for studying LCM-dissected samples with a few key advantages. For this solution hybridization assay, RNA samples (down to 10ng) were directly hybridized to the custom set of probes to generate raw counts, minimizing any bias. In contrast, microarray and RNA-seq usually require reverse transcription and/or PCR amplification of the samples before hybridization or counting. The lack of the cDNA synthesis step also allows this solution hybridization approach to tolerate some RNA degradation in samples, which is common in LCM-dissected tissue. This approach is currently not suitable for whole genome interrogation but can be used to study large numbers of genes in a single assay, making it well-suited for investigating specific molecular pathways.

The BMP system is an important regulator of cartilage development and bone growth. Bmpr1a and Bmpr1b double mutant embryos develop severe generalized chondrodysplasia and a Bmpr1a conditional knockout shows almost no long bone growth [21, 22]. We previously found a BMP expression gradient in the postnatal growth plate, with the greatest BMP ligand expressions (Bmp2 and 6) in the hypertrophic zone [8]. In the current study, we found additional evidence in support of this BMP expression gradient in the postnatal growth plate. Bmp2 and 6 were most highly expressed in the HZ, while BMP antagonists Bmp2 and Grem1 were expressed primarily in the RZ. However, immunohistochemistry of phosphorylated Smad1/5/8, which indicates active intracellular BMP signaling, showed a somewhat different pattern, with BMP signaling strongest in the PZ and prehypertrophic area and decreasing in the HZ. This suppression of BMP intracellular signaling in the HZ, despite the high expression of BMP ligands, may be attributable to upregulated expression of Smad7, in the HZ which we found both by solution hybridization and immunohistochemistry. Because Smad7 is an I-SMAD that competes for R-SMAD binding, increased Smad7 expression may help explain the decreased BMP signaling in the HZ. This hypothesis regarding the role of Smad7 in the growth plate is also consistent with recent observations in the embryonic mouse skeleton. Yoon et al found that pSmad1/5/8 abundance progressively increased from the resting zone to the prehypertrophic zone in embryonic mouse growth plates [23], and that pSmad1/5/8 levels were upregulated in the embryonic HZ in Smad7 knockout mice [24].

There is substantial evidence suggesting that the observed BMP gradients in growth plate play important roles in chondrocyte proliferation and hypertrophic differentiation. When exogenous Bmp2 was administered in a metatarsal bone culture system, moderate levels of Bmp2 promoted proliferation in the PZ, while high levels of Bmp2 stimulated hypertrophy in the HZ [10]. Similarly, when a constitutively active Bmpr1a was expressed in the cartilage, PZ height was shortened without affecting chondrocyte proliferation, suggesting excessive BMP signaling accelerates hypertrophic differentiation [11]. Furthermore, Estrada et al. showed evidence that loss of Smad7 causes impaired chondrocyte proliferation and shortening of the HZ [24], although Smad7 inhibit both BMP and TGF-β signaling and thus these growth plate phenotypes may not be solely due to disrupted BMP signaling.

Taken together, the BMP expression and functional findings suggest that active BMP signaling stimulates chondrocyte proliferation in the PZ and helps initiate hypertrophic differentiation in the prehypertrophic zone. However, the physiological importance of the apparent quenching of BMP signaling in the HZ by Smad7 remains unclear.

BMP signaling has also been shown to play a role in the maintenance of articular cartilage [25]. BMP4 and 7 have been demonstrated to promote articular cartilage repair in vitro[26, 27]. In addition, mice lacking Bmp2 in the limb element showed early postnatal fibrotic degeneration of the articular cartilage [28], while loss of Bmpr1a in the articular regions in mice showed defective joint formation and accelerated wearing of the articular cartilage with age, thereby resembling human osteoarthritis [29]. It is yet unclear whether or not the effect of BMPs on articular cartilage maintenance involves the regenerative capacity of the articular stem cells in the superficial zone. In the current study we found evidence that a similar BMP signaling gradient exists across the articular cartilage with greatest BMP action in the superficial zone. These findings are consistent with previous microarray studies that used bioinformatics to identify important pathways in spatial regulation of articular cartilage [30]. The functional consequences of this spatial expression pattern are not known. Interestingly, in articular cartilage, the greatest BMP signaling appears to occur in the superficial zone which contains progenitor cells for the articular cartilage and also provides a highly specialized surface for joint movement[7, 31]. We therefore speculate that the relatively high expression of BMP agonists in the superficial zone of the articular cartilage may, in combination with other regulatory signals, serve to maintain this highly specialized progenitor state. Consistent with this notion, expression of BMP agonists Bmp2 and 6 in the superficial zones appears to be greater than in the other zones of the articular cartilage but less than in the HZ of the growth plate, and thus BMP signaling in the SZ may be sufficient to support self-renewal but not sufficient to trigger hypertrophic differentiation [10].

In summary, we found that LCM combined with a multiplex solution hybridization assay proved to be highly effective in elucidating the spatial expression patterns for a specific pathway of interest in postnatal articular and growth plate cartilage. Using this approach, we found evidence confirming the presence of a BMP signaling gradient across the growth plate and evidence that a similar BMP gradient exists across the articular cartilage. The spatial orientation of these two signaling gradients are reversed, in that the greatest apparent BMP signaling occurs in the SZ of the articular cartilage and the PZ and prehypertrophic zone of the growth plate. Although the functional consequences are unknown, we speculate that local high BMP activity aids in the maintenance of the highly specialized progenitor population in the SZ of the articular cartilage.

## Supporting information

S1 Table nCounter data from all 6 cartilage zones(mean ±SEM) after normalization and background correction. (DOCX)

S2 Table in situ primer sequence. (DOCX)
