# Supplementary material for: Spatial regulation of bone morphogenetic proteins (BMPs) in postnatal articular and growth plate cartilage
Source: PLoS One. 2017 May 3;12(5):e0176752. doi: 10.1371/journal.pone.0176752 (PMC5414995; doi:10.1371/journal.pone.0176752)
Supplement: S1 Table — (DOCX) [file pone.0176752.s001.docx]

Table S1 nCounter data from all 6 cartilage zones(mean ±SEM) after normalization and background correction

|  | **Gene** | **SZ (mean±SEM)** | | **MZ (mean±SEM)** | | **DZ (mean±SEM)** | | **RZ (mean±SEM)** | | **PZ (mean±SEM)** | | **HZ (mean±SEM)** | |
| --- | --- | --- | --- | --- | --- | --- | --- | --- | --- | --- | --- | --- | --- |
| BMP agonist | Bmp1 | 342 | ±15 | 253 | ±14 | 274 | ±28 | 250 | ±17 | 126 | ±13 | 381 | ±60 |
|  | Bmp2 | 134 | ±15 | 37 | ±4 | 29 | ±3 | 27 | ±2 | 25 | ±0 | 359 | ±86 |
|  | Bmp4 | 31 | ±4 | 25 | ±0 | 25 | ±0 | 25 | ±0 | 25 | ±0 | 25 | ±0 |
|  | Bmp5 | 180 | ±8 | 207 | ±11 | 150 | ±24 | 129 | ±20 | 61 | ±2 | 55 | ±5 |
|  | Bmp6 | 102 | ±7 | 26 | ±1 | 25 | ±0 | 27 | ±2 | 25 | ±0 | 1369 | ±154 |
|  | Bmp7 | 25 | ±0 | 25 | ±0 | 30 | ±7 | 167 | ±19 | 142 | ±7 | 34 | ±3 |
|  | Bmp11/GDF11 | 25 | ±0 | 25 | ±0 | 25 | ±0 | 25 | ±0 | 25 | ±0 | 26 | ±1 |
|  | Bmp12/GDF7 | 25 | ±0 | 25 | ±0 | 25 | ±0 | 25 | ±0 | 25 | ±0 | 25 | ±0 |
|  | Bmp13/GDF6 | 25 | ±0 | 25 | ±0 | 25 | ±0 | 25 | ±0 | 25 | ±0 | 25 | ±0 |
|  | Bmp14/GDF5 | 32 | ±1 | 25 | ±0 | 27 | ±2 | 25 | ±0 | 25 | ±0 | 35 | ±9 |
|  | Bmp15/GDF9b | 25 | ±0 | 35 | ±5 | 42 | ±11 | 28 | ±2 | 25 | ±0 | 26 | ±0 |
| BMP antagonist | Bmp3 | 27 | ±1 | 54 | ±7 | 147 | ±25 | 268 | ±29 | 25 | ±0 | 26 | ±1 |
|  | Chord | 30 | ±3 | 25 | ±0 | 31 | ±3 | 27 | ±2 | 25 | ±0 | 25 | ±0 |
|  | Follistatin | 30 | ±2 | 42 | ±5 | 36 | ±4 | 26 | ±1 | 25 | ±0 | 27 | ±1 |
|  | Grem1 | 64 | ±10 | 408 | ±41 | 655 | ±147 | 93 | ±16 | 25 | ±0 | 25 | ±0 |
|  | Nog | 45 | ±7 | 129 | ±7 | 142 | ±19 | 201 | ±18 | 248 | ±22 | 595 | ±63 |
| BMP receptor | Bmpr1a | 304 | ±6 | 287 | ±8 | 253 | ±9 | 237 | ±22 | 248 | ±12 | 157 | ±21 |
|  | Bmpr1b | 25 | ±0 | 25 | ±0 | 26 | ±1 | 25 | ±0 | 25 | ±0 | 25 | ±0 |
|  | Bmpr2 | 168 | ±5 | 175 | ±6 | 150 | ±10 | 111 | ±10 | 110 | ±4 | 158 | ±14 |
|  | Acvr1a | 87 | ±11 | 58 | ±4 | 53 | ±6 | 52 | ±5 | 44 | ±6 | 51 | ±7 |
|  | Acvr1b | 53 | ±5 | 102 | ±10 | 92 | ±22 | 104 | ±14 | 62 | ±5 | 63 | ±9 |
|  | Acvr2a | 42 | ±2 | 45 | ±5 | 58 | ±9 | 40 | ±5 | 26 | ±1 | 25 | ±0 |
|  | Acvr2b | 39 | ±1 | 63 | ±4 | 46 | ±4 | 38 | ±6 | 28 | ±1 | 38 | ±6 |
|  | Acvrl1 | 46 | ±8 | 50 | ±5 | 36 | ±6 | 29 | ±2 | 26 | ±1 | 32 | ±4 |
| SMADs | Smad1 | 82 | ±5 | 88 | ±4 | 72 | ±10 | 101 | ±4 | 129 | ±7 | 125 | ±9 |
|  | Smad4 | 122 | ±4 | 112 | ±10 | 119 | ±6 | 99 | ±6 | 82 | ±4 | 93 | ±3 |
|  | Smad5 | 273 | ±6 | 313 | ±10 | 273 | ±13 | 292 | ±12 | 175 | ±10 | 159 | ±18 |
|  | Smad6 | 66 | ±5 | 63 | ±8 | 42 | ±8 | 32 | ±3 | 39 | ±10 | 38 | ±6 |
|  | Smad7 | 31 | ±1 | 35 | ±3 | 27 | ±2 | 26 | ±1 | 25 | ±0 | 82 | ±7 |
|  | Smad8 | 29 | ±3 | 47 | ±3 | 34 | ±6 | 25 | ±0 | 26 | ±1 | 34 | ±5 |
| Zonal marker | Prg4 | 4973 | ±458 | 204 | ±59 | 29 | ±5 | 25 | ±0 | 25 | ±0 | 25 | ±0 |
|  | Sfrp5 | 59 | ±6 | 169 | ±21 | 324 | ±44 | 482 | ±130 | 82 | ±8 | 37 | ±5 |
|  | Gdf10 | 189 | ±15 | 50 | ±6 | 34 | ±4 | 180 | ±37 | 1695 | ±71 | 262 | ±112 |
|  | Prelp | 1212 | ±119 | 366 | ±35 | 282 | ±80 | 1080 | ±125 | 2493 | ±224 | 305 | ±117 |
|  | Col10a1 | 80 | ±123 | 98 | ±21 | 128 | ±56 | 141 | ±262 | 166 | ±49 | 29917 | ±2726 |
| Housekeeping genes | Actb | 3973 | ±527 | 2698 | ±180 | 2563 | ±264 | 2421 | ±256 | 2287 | ±212 | 3525 | ±348 |
|  | Col2a1 | 172846 | ±18633 | 328555 | ±18327 | 235481 | ±32084 | 129900 | ±18711 | 102177 | ±5997 | 94111 | ±12987 |
|  | Hmgn1 | 412 | ±25 | 472 | ±27 | 437 | ±13 | 365 | ±8 | 326 | ±6 | 354 | ±17 |
|  | Rpl13a | 184 | ±10 | 204 | ±5 | 255 | ±14 | 304 | ±9 | 411 | ±11 | 304 | ±9 |
|  | Rpl35 | 3603 | ±152 | 4186 | ±154 | 3804 | ±218 | 4047 | ±319 | 3538 | ±241 | 2862 | ±205 |
