# Supplementary material for: Spatial regulation of bone morphogenetic proteins (BMPs) in postnatal articular and growth plate cartilage
Source: PLoS One. 2017 May 3;12(5):e0176752. doi: 10.1371/journal.pone.0176752 (PMC5414995; doi:10.1371/journal.pone.0176752)
Supplement: S2 Table — (DOCX) [file pone.0176752.s002.docx]

Table S2 in situ primer sequence

| Gene | Forward primer | Reverse primer | Amplicon |
| --- | --- | --- | --- |
| Bmp2 | 5’ CCCCAGACCACCGGCT 3’ | 5’ GGTTCTTATCCAAATATTCCCCCTA 3’ | 1577bp |
| Bmp3 | 5’ TTACTTCTGCCTGAACCTGGC 3’ | 5’ CCACCCAATCTTAAAGCCCAA 3’ | 1680bp |
| Bmp6 | 5’ GCGCTGCACACTCCTTGA 3’ | 5’ AATCCAAGGCAGAACCATGC 3’ | 1060bp |
| Gdf10 | 5’ CTGAAGCGAGATTGGTGCAAA 3’ | 5’ CATAGGGCGAGCACTGAACAG 3’ | 1000bp |
| Grem1 | 5’ GGACATGGTCGCTATCCACAT 3’ | 5’ GTAGGCGTCAAAGGATTTGGG 3’ | 705bp |
